# Copper Concentrations in Ketamine Therapy for Treatment-Resistant Depression

**DOI:** 10.3390/brainsci10120971

**Published:** 2020-12-11

**Authors:** Jakub Słupski, Wiesław Jerzy Cubała, Natalia Górska, Anita Słupska, Maria Gałuszko-Węgielnik

**Affiliations:** Department of Psychiatry, Faculty of Medicine, Medical University of Gdańsk, 80-952 Gdansk, Poland; cubala@gumed.edu.pl (W.J.C.); nataliagorska@gumed.edu.pl (N.G.); slupskaanita@gumed.edu.pl (A.S.); mgaluszko@gumed.edu.pl (M.G.-W.)

**Keywords:** copper, ketamine, treatment-resistant mood disorders

## Abstract

Changes in serum copper concentration are observed in patients with depressive symptoms. Unmet needs in contemporary antidepressant treatment have increased interest in non-monoaminergic antidepressants, such as ketamine, an anaesthetic drug that has demonstrated a rapid antidepressant effect in patients with treatment-resistant depression (TRD). The purpose of this study was to examine whether serum copper concentrations change during ketamine treatment and whether there is an association between the copper concentrations and treatment response measured using psychometric scale scores. Moreover, the interlink between somatic comorbidities and copper concentration was studied. Patients with major depressive disorder or bipolar disorder were rated weekly by a clinician using the Montgomery–Asberg Depression Rating Scale (MADRS) and Young Mania Rating Scale (YMRS). Copper level assessments were carried out weekly before the start of ketamine treatment and then after every second infusion and one week after the last ketamine infusion. The serum concentration of copper before ketamine treatment was significantly higher than that after the fifth infusion (*p* = 0.016), and the serum concentration after the treatment was significantly higher than that after the fifth infusion (*p* = 0.048). No significant correlations between changes in the copper serum concentrations and MADRS or YMRS were found. The serum copper level was not associated with somatic comorbidities during the course of treatment. This study provides data on the role of copper in short-term intravenous ketamine treatment in TRD, although no clear evidence of a connection between the copper level and treatment response was found.

## 1. Introduction

Treatment-resistant depression (TRD), which can occur in both major depressive disorder (MDD) and bipolar disorder (BP), remains an important psychiatric issue as a number of patients which do not attain remission after being treated following the contemporary standard of care (SOC) [1,2].

Unmet needs in contemporary antidepressant treatment have increased interest in non-monoaminergic antidepressants, such as ketamine, an anaesthetic drug that demonstrates antidepressive action in patients with treatment-resistant depression (TRD) [3,4] and is thus used in psychiatry to promote fast-acting antidepressant and anti-suicidal effects [5]. Considering the enhanced response compared to that to conventional antidepressant treatment, ketamine, an N-methyl-D-aspartate receptor (NMDAR) antagonist, seems to be a promising drug to treat TRD [6]. Divalent ions, such as copper, are engaged in the function and homeostasis of neurotransmission systems [7]. Recent studies have shown that abnormal copper levels might be associated with depressive symptoms [8]; moreover, copper plays an important role in the regulation of the NMDAR and the α-amino-3-hydroxy-5-methyl-4isoxazole-propionic acid receptor (AMPAR) [9]. Copper also interacts with dopamine β-hydroxylase, which is a member of a class of copper-containing hydroxylases, playing a vital role in the biosynthesis of neurotransmitters [10]. Furthermore, copper was found to be a potent monoamine oxidase type A and B (MAO-A and MAO-B, respectively) inhibitor, and these enzymes play a key role in dopamine, norepinephrine, and serotonin degradation [11,12]. Although the hypothesis of interlinkage between copper, ketamine action, and TRD management has little evidence to date, the elaboration of that potential connection is of interest [6].

Considering the contribution of the interaction between copper and NMDAR to TRD, ketamine appears to address vital facets of TRD phenomena, exhibiting rapid antidepressive efficacy. The aim of this study was to investigate the associations between blood copper concentration and psychometric measures in patients with TRD during the course of MDD and BP.

## 2. Materials and Methods

The study population included individuals enrolled in a naturalistic observational registry protocol for ketamine intravenous infusions given to treat TRD (NCT04226963), which has previously been described in detail elsewhere [13,14]. Briefly, the inpatients diagnosed with depressive episodes during the course of MDD or BP were included. The diagnosis was established by a clinician psychiatrist according to the Diagnostic and Statistical Manual of Mental Disorders, 5th edition criteria using the Mini International Neuropsychiatric Interview. All participants demonstrated treatment resistance for the existing depressive episode. Single-patient and single-rater rules were followed during the study. Only medically stable individuals were enrolled in the study. Ketamine was administered along with SOC psychotropic medication.

This dataset represents the intermittent analysis of an observational study performed for the interim modelling of observational learning. The analyses are set per predefined and equally spaced annual increments for a study duration of 24 months with a population of 120 TRD subjects estimated for inclusion. This study was conducted in accordance with the latest version of the Declaration of Helsinki. The study protocol was approved by the Independent Ethics Committee of the institution (NKBBN/172/2017; 172-674/2019). All study participants provided written consent for participation in the study.

### 2.1. Study Design

This study used an observational design with the administration of eight ketamine intravenous infusions over 4 weeks in TRD subjects. We followed the treatment-resistance definition as an insufficient response to at least two trials of treatment [15]. Ketamine was dosed at 0.5 mg/kg based on the patient’s actual body weight and infused intravenously over 40 min.

Safety monitoring was scheduled before, during, and after infusion every 15 min, up to 90 min post infusion, and including the periodic assessment of vital signs (heart rate, body temperature, respiratory rate, blood pressure, and oxygen saturation). Psychometric assessment using the Montgomery–Asberg Depression Rating Scale (MADRS) and Young Mania Rating Scale (YMRS) was performed before the first, third, fifth, and seventh infusions and one week after the last infusion.

Participants were defined as responders at a given time point if the percent improvement from baseline in the MADRS total score was at least 50%; on the contrary, they were defined as remitters at a given time point if the MADRS total score was ≤10 points [16].

Copper levels were assessed on a weekly basis, before every second infusion, and one week after the last ketamine infusion. Serum copper ion concentrations were determined using a direct, colorimetric measurement method using a commercially available two-reagent 6K93-30 MULTIGENT Copper kit (SENTINEL CH. SpA, Italy) with a detection limit of 3 µg/dL (0.47 µmol/L). The blood was sampled for heparin, immediately centrifuged at 4000 rpm, and the serum was transferred and sent for assay.

### 2.2. Statistics

The Shapiro–Wilk test was used to assess the distribution of continuous data. Normally distributed variables were compared using the Student’s t-test, and all other continuous data were compared using the nonparametric Mann–Whitney U-test. All tests were two-tailed with an alpha value of 0.05. The Wilcoxon rank test was used to analyse the serum copper concentration, diagnosis, MADRS, YMRS, and comorbidities. To analyse the dynamics of changes in serum copper and psychometric variables during ketamine infusions, repeated measures of analysis of variance were performed. No sphericity was found using the Mauchly test (*p* < 0.05); therefore, the Greenhouse–Geisser correction was used in the analyses. To determine which measurements had significant differences, a post hoc analysis was performed using the Bonferroni test.

## 3. Results

**Figure 1:**
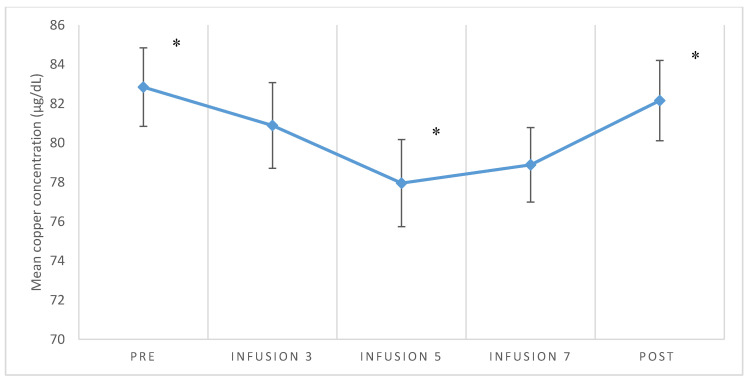
Mean values and standard errors of the mean values for the concentration of serum copper ions in individual measurements; * the concentration of serum copper in the pre-treatment measurement was significantly higher than that after the fifth infusion (*p* = 0.016), and the concentration after the treatment was significantly higher than that after the fifth infusion (*p* = 0.048).

The demographics and clinical characteristics of the study group are presented in Table 1. Values in brackets represent the percentage of the total number of participants (*n* = 49). The concentration of serum copper varied significantly depending on the measurement (F (3.029) = 4.214; *p* = 0.007; η^2^_p_ = 0.09). The concentration of serum copper before treatment with ketamine was significantly higher than that after the fifth infusion (*p* = 0.016) and the concentration after treatment was significantly higher than that after the fifth infusion (*p* = 0.048). The differences in the serum copper levels between other measurements were non-significant. Figure 1 illustrates the mean values with standard errors of the mean. 

**Figure 2:**
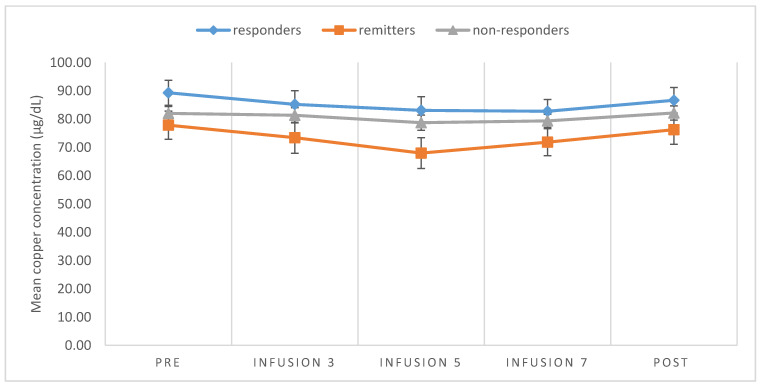
Mean values and standard errors for changes in copper serum levels among responders, remitters, and non-responders.

For TRD in both patients with MDD and BP, the copper concentration did not significantly change during the treatment (Figure 1). In addition, the change in copper in relation to the MADRS score as well as the clinical response, defined as the responder, remitter, and non-responder, turned out to be non-significant during the course of treatment (Figure 2). There was no significant correlation between changes in the serum copper concentration and MADRS or YMRS.

The concentration of copper during the course of treatment was not associated with somatic comorbidities (Table 2); however, we found that arterial hypertension was significantly more common in the group of responders, while diabetes mellitus occurred significantly more often in the group of remitters (Table 1).

## 4. Discussion

Serum copper concentrations during the course of treatment with intravenous ketamine given in addition to SOC to treat TRD did not change after the end of the intervention, regardless of the treatment outcome or psychometric measures. Somatic comorbidities did not affect the copper concentrations in the study population. The reason behind the transitional decrease in copper concentration during the ketamine treatment remains largely unclear; however, this is consistent with the finding of sertraline-induced copper decrease in patients with MDD [7]. Thus, the intermittent decrease in copper concentration may be associated with a reduction in the acute phase response, possibly induced by ketamine. 

Studies on copper levels in patients with MDD have produced inhomogeneous results. Our study agrees with previous data from a study of a homogeneous MDD population [17], indicating no correlation between copper ion concentrations and the stage of the disease or the treatment outcome. Liu et al. [18] found that there were no significant correlations between the depression severity scores (based on the Hamilton Depression Scale-24 version) or disease course and abnormal copper levels in patients with MDD. However, another study [19] demonstrated that children and adolescents with moderate and severe depression had significantly higher copper levels. The evaluation of results becomes more difficult considering the studies reporting both lower copper levels in depression [17] and a significant increase in serum copper levels compared with that in controls [20]. Moreover, the interpretation of studies that did not control for overt inflammation is even more challenging, as overt inflammation coexists with increased levels of ceruloplasmin, an acute phase protein that transports copper [7]. There is plentiful confounding data on the factors affecting copper concentrations, including a correlation between sertraline use and lower copper concentration [7]. In an animal study using brain microdialysis in mice exposed to chelators, copper levels were significantly increased in the striatum. According to the authors, this might contribute to neurological deterioration [21]. However, neurological abnormalities occur in copper-deficient blotchy mutant mice (MoB), which have genetic defects of the copper-transporting ATPase gene [22]. In addition, animals with zinc-related genetic deficiencies develop symptoms related to zinc deficiency, including neuropsychiatric changes, loss of appetite, and irritability [23].

Study findings remain inconsistent, and the role of copper ions in ketamine treatment has been questioned. The divergent findings may be related to methodological differences, including the selection criteria for illness chronicity, sample sizes, unmatched groups, age, sex, concomitant medication, and the choice of antidepressant therapy. Furthermore, copper is a vital element in the inhibition of NMDAR channels [24]. Copper is involved in oxidative stress processes. This influences the catalytic and structural properties of antioxidant enzymes, which may be among the main causes of the development of depression [25,26]. Some studies suggest that the induction of oxidative stress pathways in depression is accompanied by the activation of the inflammatory reaction [27]. Processes in which copper plays important roles include catecholamine metabolism; the functioning of NMDA, α-amino-3-hydroxy-5-methyl-4-isoxazolepropionic acid (AMPA), gamma-aminobutyric acid (GABA), glycine, and kainate receptors; neurogenesis; synaptogenesis; learning and memory function; antioxidant processes; and regulation of the immunological system [28,29,30,31]. Copper plays an important role in the transformation of dopamine into norepinephrine, as copper ions interact with dopamine β-hydroxylase [32,33]. A possible connection between copper concentrations and ketamine treatment may be the inhibition of NMDARs by extracellular magnesium ions when a negative membrane potential is present [34]. Zinc ions also bind to NMDARs to modulate their activity [35]. Khosravani et al. [36] and You et al. [37] revealed that NMDARs are also regulated by the cellular prion protein (PrP^C^). Furthermore, the NMDA-irrelevant ketamine mechanism of action has been identified in animal studies, as ketamine induces the pairing of Gαs and adenylyl cyclase, and through this mechanism, increases the intracellular cyclic adenosine monophosphate (cAMP), which affects the phosphorylation of cAMP response element-binding protein (CREB), increasing BDNF expression [38]. 

## 5. Limitation

There are several limitations to this study. This study was conducted using a small sample, without randomisation, and an inactive placebo comparison, without a control group. There was no separation by diagnosis; thus, the findings of the study apply to TRD patients with MDD and BP. Nevertheless, nonhomogeneous populations comprising MDD and BP patients, with or without comorbidities represent real-world clinical practice. Another limitation of this study is the lack of measurements of ketamine and its metabolites in the blood. Moreover, we did not measure copper levels in the central spinal fluid; instead, we measured serum copper levels. There was no measurement of ceruloplasmin serum levels, although according to Kaya et al. [39], no noteworthy alterations in serum ceruloplasmin levels were found in patients with improved clinical measures of depression after antidepressant treatment. Finally, multiple confounding factors (e.g., nutritional status, limit of detection) could have influenced our results.

## 6. Conclusions

The present study does not support the hypothesis of significant changes in copper concentration in ketamine intravenous add-on treatment, signalling the possible interlink between ketamine administration and copper concentration in the active phase of treatment.

## Figures and Tables

**Table 1:** Demographics and clinical profile.

			*N*	Responder	Remitter	Non-Responder	*p*	V
Male sex	(%)		21 (42.9)	6 (66.7)	2 (25.0)	13 (40.6)	0.229	0.26
Female sex	(%)		28 (57.1)	3 (33.3)	6 (75.0)	19 (59.4)		
Mean age, in years			50.02	53.11	42.88	50.94	0.336	0.00
Occupation							0.250	0.32
	Employed		11 (22.4)	2 (22.2)	2 (25.0)	7 (21.9)		
	Unemployed		27 (55.1)	7 (77.8)	3 (37.5)	18 (56.2)		
	Pension		10 (20.4)	0 (0)	3 (37.5)	7 (21.9)		
	Seasonal work		1 (2.0)	0 (0)	0 (0)	1 (3.1)		
Education							0.252	0.26
	Elementary or lower		2 (4.1)	0 (0)	1 (12.5)	1 (3.1)		
	Vocational		5 (10.2)	2 (22.2)	1 (12.5)	2 (6.3)		
	Secondary		18 (36.7)	1 (11.1)	3 (37.5)	14 (43.8)		
	Higher		24 (49.0)	6 (66.7)	3 (37.5)	15 (46.9)		
BMI			27.92 (5.67)	28.00 (4.64)	26.50 (4.72)	28.25 (6.21)	0.613	0.02
Ketamine treatment for:								
	MDD		35 (71.4)	8 (88.9)	5 (62.5)	22 (68.8)	0.475	0.19
	BP		14 (28.6)	2 (11.1)	5 (37.5)	7 (31.2)	0.485	0.18
Comorbidity							0.104	0.31
		1	21 (42.9)	6 (66.7)	2 (25.0)	13 (40.6)		
		2	10 (20.4)	2 (22.2)	1 (12.5)	7 (21.9)		
		3	4 (8.2)	1 (11.1)	2 (25.0)	1 (3.1)		
	Arterial hypertension		16 (32.7)	6 (66.7)	3 (37.5)	7 (21.9)	0.037	0.37
		BP	4 (8.2)	1 (11.1)	2 (25.0)	1 (3.1)	0.052	0.66
		MDD	12 (24.5)	5 (55.6)	1 (12.5)	6 (18.8)	0.177	0.33
	Diabetes mellitus		3 (6.1)	1 (11.1)	2 (25.0)	0 (0)	0.021	0.39
	Hyperlipidaemia		9 (18.4)	3 (33.3)	1 (12.5)	5 (15.6)	0.545	0.19
	Post-stroke		3 (6.1)	1 (11.1)	0 (0)	2 (6.3)	0.731	0.14
	Post-myocardial infarction		0 (0)	0 (0)	0 (0)	0 (0)	-	-
	Epilepsy		6 (12.2)	0 (0)	3 (37.5)	3 (9.4)	0.060	0.36
	Other		16 (32.7)	2 (22.2)	1 (12.5)	13 (40.6)	0.330	0.24
Coexisting treatment								
	TCA		8 (16.3)	1 (11.1)	1 (13.5)	6 (18.8)	1.000	0.09
	Clomipramine		4 (8.2)	0 (0)	1 (12.5)	3 (9.4)	0.789	0.15
	Amitriptyline		4 (8.2)	1 (11.1)	0 (0)	3 (9.4)	1.000	0.13
	SSRI total		23 (46.9)	5 (55.6)	2 (25.0)	16 (50.0)	0.413	0.20
	Fluvoxamine		1 (2.0)	0 (0)	0 (0)	1 (3.1)	1.000	0.11
	Paroxetine		5(10.2)	1 (11.1)	0 (0)	4 (12.5)	0.813	0.15
	Fluoxetine		8 (16.3)	2 (22.2)	0 (0)	6 (18.8)	0.534	0.20
	Sertraline		3 (6.1)	1 (11.1)	0 (0)	2 (6.3)	0.731	0.14
	Citalopram		4 (8.2)	0 (0)	2 (25.0)	2 (6.3)	0.179	0.29
	Escitalopram		2 (4.1)	1 (11.1)	0 (0)	1 (3.1)	0.578	0.18
	SNRI total		11 (22.4)	2 (22.2)	2 (25.0)	7 (21.9)	1.000	0.03
	Venlafaxine		8 (16.3)	1 (11.1)	1 (12.5)	6 (18.8)	1.000	0.10
	Duloxetine		3 (6.1)	1 (11.1)	1 (12.5)	1 (3.1)	0.273	0.17
	Other ADTs:						0.749	0.14
		1	15 (30.6)	4 (44.4)	2 (25.0)	9 (28.1)		
		2	3 (6.1)	0 (0)	1 (12.5)	2 (6.3)		
	Mirtazapine		9 (18.4)	2 (22.2)	1 (12.5)	6 (18.8)	1.000	0.08
	Mianserin		3 (6.1)	1 (11.1)	0 (0)	2 (6.3)	0.731	0.14
	Trazodone		4 (8.2)	1 (11.1)	1 (12.5)	2 (6.3)	0.432	0.10
	Bupropion		3 (6.1)	0 (0)	1 (12.5)	2 (6.3)	0.488	0.15
	Vortioxetine		2 (4.1)	0 (0)	1 (12.5)	1 (3.1)	0.333	0.20
	Antipsychotics						0.806	0.15
		1	12 (24.5)	2 (22.2)	1 (12.5)	9 (28.1)		
		2	5 (10.2)	0 (0)	1 (12.5)	4 (12.5)		
	Aripiprazole		6 (12.2)	0 (0)	1 (12.5)	5 (15.6)	0.685	0.18
	Quetiapine		10 (20.4)	1 (11.1)	1 (12.5)	8 (25.0)	0.668	0.16
	Olanzapine		5 (10.2)	1 (11.1)	1 (12.5)	3 (9.4)	1.000	0.04
	Risperidone		1 (2.0)	0 (0)	0 (0)	1 (3.1)	1.000	0.11
	Mood stabilisers						0.348	0.29
		1	15 (30.6)	2 (22.2)	4 (50.0)	9 (28.1)		
		2	6 (12.2)	1 (11.1)	0 (0)	5 (15.6)		
		3	1 (2.0)	0 (0)	1 (12.5)	0 (0)		
	Lithium		5 (10.2)	0 (0)	1 (12,5)	4 (12.5)	0.643	0.16
	Valproate		9 (18.4)	2 (22.2)	3 (37.5)	4 (12.5)	0.160	0.24
	Lamotrigine		7 (14.3)	1 (11.1)	1 (12.5)	5 (15.6)	1.000	0.05

N, sample size; *p*, probability value; V, Cramer’s V; MDD, major depressive disorder; BP, bipolar disorder; TCA, other tricyclic antidepressant; SSRI, selective serotonin reuptake inhibitor; SNRI, selective serotonin-noradrenaline reuptake inhibitors; ADTs, antidepressants; BMI, body mass index.

**Table 2:** Serum copper concentration versus comorbidity.

Comorbidity	F	df	*p*	η^2^_p_
No. of comorbidities	0.65	9.12	0.757	0.05
Arterial hypertension	0.42	3.04	0.743	0.01
Diabetes mellitus	1.18	3.02	0.322	0.03
Hyperlipidaemia	1.05	3.00	0.371	0.02
Stroke	1.32	3.06	0.272	0.03
Epilepsy	0.44	3.01	0.723	0.01
Other	0.97	3.04	0.409	0.02

## References

[1] Gelenberg: A.J. (2010). The prevalence and impact of depression. J. Clin. Psychiatry.

[2] McIntyre R.S., Filteau M.-J., Martin L., Patry S., Carvalho A., Cha D.S., Barakat M., Miguelez M. (2014). Treatment-resistant depression: Definitions, review of the evidence, and algorithmic approach. J. Affect. Disord..

[3] Diamond P.R., Farmery A.D., Atkinson S., Haldar J., Williams N., Cowen P.J., Geddes J.R., McShane R. (2014). Ketamine infusions for treatment resistant depression: A series of 28 patients treated weekly or twice weekly in an ECT clinic. J. Psychopharmacol..

[4] McGirr A., Berlim M.T., Bond D.J., Fleck M.P., Yatham L.N., Lam R.W. (2015). A systematic review and meta-analysis of randomized, double-blind, placebo-controlled trials of ketamine in the rapid treatment of major depressive episodes. Psychol. Med..

[5] Larkin G.L., Beautrais A.L. (2011). A preliminary naturalistic study of low-dose ketamine for depression and suicide ideation in the emergency department. Int. J. Neuropsychopharmacol..

[6] Słupski J., Cubała W.J., Górska N., Gałuszko-Węgielnik M., Wiglusz M.S. (2018). Role of copper in depression. Relationship with ketamine treatment. Med. Hypotheses.

[7] Twayej A.J., Al-Hakeim H.K., Al-Dujaili A.H., Maes M. (2020). Lowered zinc and copper levels in drug-naïve patients with major depression: Effects of antidepressants, ketoprofen and immune activation. World J. Biol. Psychiatry.

[8] Ni M., You Y., Chen J., Zhang L. (2018). Copper in depressive disorder: A systematic review and meta-analysis of observational studies. Psychiatry Res..

[9] Huang S., Chen L., Bladen C., Stys P.K., Zamponi G.W. (2018). Differential modulation of NMDA and AMPA receptors by cellular prion protein and copper ions. Mol. Brain.

[10] Vendelboe T.V., Harris P., Zhao Y., Walter T.S., Harlos K., El Omari K., Christensen H.E.M. (2016). The crystal structure of human dopamine β-hydroxylase at 2.9 Å resolution. Sci. Adv..

[11] Magour S., Cumpelik O., Paulus M. (1979). Effect of Cadmium and Copper on Monoamine Oxidase Type A and B in Brain and Liver Mitochondria. Clin. Chem. Lab. Med..

[12] Whibley A.C., Urquhart J., Dore J., Willatt L.R., Parkin G., Gaunt L., Black G., Donnai D., Raymond F.L. (2010). Deletion of MAOA and MAOB in a male patient causes severe developmental delay, intermittent hypotonia and stereotypical hand movements. Eur. J. Hum. Genet..

[13] Słupski J., Cubała W.J., Górska N., Słupska A., Gałuszko-Węgielnik M. (2020). Copper and anti-anhedonic effect of ketamine in treatment-resistant depression. Med. Hypotheses.

[14] Szarmach J., Cubała W.J., Włodarczyk A., Gałuszko-Węgielnik M. (2020). Metabolic Risk Factors and Cardiovascular Safety in Ketamine Use for Treatment Resistant Depression. Neuropsychiatr. Dis. Treat..

[15] Voineskos D., Daskalakis Z.J., Blumberger D.M. (2020). Management of treatment-resistant depression: Challenges and strategies. Neuropsychiatr. Dis. Treat..

[16] Trivedi M.H., Corey-Lisle P.K., Guo Z., Lennox R.D., Pikalov A., Kim E. (2009). Remission, response without remission, and nonresponse in major depressive disorder: Impact on functioning. Int. Clin. Psychopharmacol..

[17] Styczeń K., Sowa-Kućma M., Siwek M., Dudek D., Reczyński W., Misztakk P., Szewczyk B., Topór-Mądry R., Opoka W., Nowak G. (2016). Study of the Serum Copper Levels in Patients with Major Depressive Disorder. Biol. Trace Element Res..

[18] Liu X., Zhong S., Li Z., Chen J., Wang Y., Lai S., Miao H., Jia Y. (2020). Serum copper and zinc levels correlate with biochemical metabolite ratios in the prefrontal cortex and lentiform nucleus of patients with major depressive disorder. Prog. Neuro Psychopharmacol. Biol. Psychiatry.

[19] Alghadir A.H., Gabr S.A., Al-Eisa E. (2015). Effects of Physical Activity on Trace Elements and Depression Related Biomarkers in Children and Adolescents. Biol. Trace Element Res..

[20] Islam R., Islam R., Qusar M.M.A.S., Islam M.S., Kabir H., Rahman G.K.M.M., Islam S., Hasnat A. (2018). Alterations of serum macro-minerals and trace elements are associated with major depressive disorder: A case-control study. BMC Psychiatry.

[21] Zhang J.-W., Liu J.-X., Hou H.-M., Chen D.-B., Feng L., Wu C., Ji-Wei Z., Li-Ting W. (2015). Effects of tetrathiomolybdate and penicillamine on brain hydroxyl radical and free copper levels: A microdialysis study in vivo. Biochem. Biophys. Res. Commun..

[22] Bull R.J., Aschner M., Brewer G.J., Harris E.D., Keen C.L., Sunderman F.W., Tsuji J.S., Zeise L.A. (2000). National Research Council (US) Committee on Copper in Drinking Water Health Effects of Excess Copper.

[23] Ackland L., Michalczyk A. (2006). Zinc deficiency and its inherited disorders—A review. Genes Nutr..

[24] Słupski J., Słupska A., Szałach Ł.P., Włodarczyk A., Górska N., Szarmach J., Jakuszkowiak-Wojten K., Gałuszko-Węgielnik M., Wilkowska A., Wiglusz M.S. (2019). Role of copper and ketamine in major depressive disorder—An update. Proc. Psychiatr. Danub..

[25] Collins J.F., Prohaska J.R., Knutson M.D. (2010). Metabolic crossroads of iron and copper. Nutr. Rev..

[26] Młyniec K., Gaweł M., Doboszewska U., Starowicz G., Pytka K., Davies C.L., Budziszewska B. (2015). Essential elements in depression and anxiety. Part II. Pharmacol. Rep..

[27] Maes M. (2011). Depression is an inflammatory disease, but cell-mediated immune activation is the key component of depression. Prog. Neuro Psychopharmacol. Biol. Psychiatry.

[28] Maret W., Sandstead H.H. (2006). Zinc requirements and the risks and benefits of zinc supplementation. J. Trace Elem. Med. Biol..

[29] Nowak G. (2015). Zinc, future mono/adjunctive therapy for depression: Mechanisms of antidepressant action. Pharmacol. Rep..

[30] Szewczyk B. (2013). Zinc homeostasis and neurodegenerative disorders. Front. Aging Neurosci..

[31] Maes M., Vandoolaeghe E., Neels H., Demedts P., Wauters A., Meltzer H.Y., Altamura C., DeSnyder R. (1997). Lower serum zinc in major depression is a sensitive marker of treatment resistance and of the immune/inflammatory response in that illness. Biol. Psychiatry.

[32] Rahman M.K., Rahman F., Rahman T., Kato T. (2009). Dopamine-ß-hydroxylase (DBH), its cofactors and other biochemical parameters in the serum of neurological patients in Bangladesh. Int. J. Cardiol..

[33] Crayton J.W., Walsh W.J. (2007). Elevated serum copper levels in women with a history of post-partum depression. J. Trace Elements Med. Biol..

[34] Nowak L., Bregestovski P., Ascher P., Herbet A., Prochiantz A. (1984). Magnesium gates glutamate-activated channels in mouse central neurones. Nat. Cell Biol..

[35] Peters S., Koh J., Choi D.W. (1987). Zinc selectively blocks the action of N-methyl-D-aspartate on cortical neurons. Science.

[36] Khosravani H., Zhang Y., Tsutsui S., Hameed S., Altier C., Hamid J., Chen L., Villemaire M., Ali Z., Jirik F.R. (2008). Prion protein attenuates excitotoxicity by inhibiting NMDA receptors. J. Cell Biol..

[37] You H., Tsutsui S., Hameed S., Kannanayakal T.J., Chen L., Xia P., Engbers J.D.T., Lipton S.A., Stys P.K., Zamponi G.W. (2012). A neurotoxicity depends on interactions between copper ions, prion protein, and N-methyl-D-aspartate receptors. Proc. Natl. Acad. Sci. USA.

[38] Czysz A.H., Schappi J.M., Rasenick M.M. (2015). Lateral Diffusion of Gαs in the Plasma Membrane Is Decreased after Chronic but not Acute Antidepressant Treatment: Role of Lipid Raft and Non-Raft Membrane Microdomains. Neuropsychopharmacology.

[39] Kaya M.C., Bez Y., Selek S., Karababa I.F., Bulut M., Savaş H.A., Çelik H., Herken H. (2012). No Effect of Antidepressant Treatment on Elevated Serum Ceruloplasmin Level in Patients with First-Episode Depression: A Longitidunal Study. Arch. Med Res..

